# A Review and Summary of Patients with Symptomatic Postoperative Discal Pseudocysts of the Lumbar Spine

**DOI:** 10.1111/os.13689

**Published:** 2023-03-31

**Authors:** Zhenlu Cao, Yanan Cong, Chuqiang Yin, Yuelei Wang, Zhichao Wang, XiaoWei Liu, Ting Wang

**Affiliations:** ^1^ Department of Orthopaedic Surgery The Affiliated Hospital of Qingdao University Qingdao China; ^2^ Department of Health Management Center The Affiliated Hospital of Qingdao University Qingdao China

**Keywords:** Discal pseudocyst, Lumbar disc herniation, Postoperative complication, Surgical

## Abstract

**Objective:**

Postoperative discal pseudocyst (PDP) is a rare complication after discectomy. This study aimed to summarize the characteristics, pathological mechanisms and management of PDPs.

**Methods:**

Nine patients with PDP who received surgical treatment at our institution from January 2014 to December 2021 were retrospectively reviewed. A systematic review of the literature on PDP was performed. The demographic data, clinical and imaging features, surgical options and patient prognosis were analyzed.

**Results:**

Among the nine patients treated at our center, seven were male and two were female. The mean patient age (± standard deviation) at the time of surgery was 28.3 ± 5.7 years (range 18–37 years). The first operation performed on seven patients was percutaneous endoscopic transforaminal discectomy (PETD) and two patients underwent microdiscectomy. The time to conservative treatment before surgical intervention was 20 ± 9.2 days. In three cases, the disc cysts were located in L4/5 and in six cases the lesions were located in L5/S1. Intervertebral disc cyst interventions included foraminal scope (three cases), open discectomy (three cases), conservative treatment with a quadrant channel (one case) and CT‐guided puncture (one case). All patients fully recovered after surgery and the mean follow‐up time was 3.5 ± 2.1 years. A literature review identified 14 relevant articles that reported 43 PDP cases of PDP.

**Conclusion:**

PDP occurs in Asian males with mild intervertebral disc degeneration and occurs 1 month after discectomy. Treatment should be based on specific patient scenarios. Conservative treatment is necessary and surgery should be performed with caution.

## Graphical abstract

A systematic literature review been performed about Postoperative Discal Pseudocyst (PDP). Demographic data, clinical features, imaging features, surgical options and prognosis were analyzed.

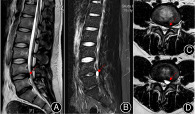

## Introduction

Lumbar disc herniation (LDH) is a common condition that usually requires surgery with the aim to improve physical function.^1^ Recently, microsurgery or endoscopic discectomy has become the main option for patients with LDH as it is minimally invasive and has a fast postoperative recovery rate.^2^ However, the complications remain relatively common and include recurrent disc herniation, incomplete disc removal, nerve root injury, epidural tear, epidural hematoma and nerve root artery injury.^3^, ^4^

In addition, postoperative discal pseudocyst (PDP) is a rare complication associated with endoscopic discectomy that was first reported by Young *et al*.^5^ A pseudocyst is a cystic lesion at the site of disc surgery that compresses the nerve root and often results in a recurrence or exacerbation of symptoms before surgery.^5^, ^6^ The morphology and imaging manifestations of pseudocysts are similar to intervertebral disc cysts. However, due to the incomplete structure of the PDP capsule wall, the lesions are called pseudocysts. Pseudocysts are generally found during the early postoperative MRI examinations of patients showing a surgical area of annulus fibrosus and protruding annulus fibrosus on the surface. T2‐weighted images with a high signal and T1‐weighted images with a low signal show that the lesions can penetrate the intervertebral foramen or spinal canal.^7^ Advances in imaging techniques have improved the diagnosis of pseudocysts, however, as intervertebral disc cysts are very rare, the characteristics and associated mechanisms of the disease are yet to be fully understood.

Here, we report nine cases of symptomatic PDP and conducted a systematic literature review to summarize the characteristics of PDP and current treatment strategies. We present a summary of our clinical experiences of PDP to improve the understanding of the disease based on reported evidence.

## Methods

Data from nine patients with PDP who underwent surgical treatment at our center from January 2014 to December 2021 were retrospectively analyzed. All of these patients had recurrent symptoms including lumbar and leg pain after minimally invasive discectomy and had the general characteristics of intervertebral disc cysts proposed by Chiba.^8^ The clinical data including general information, natural history, clinical manifestations, radiological manifestations, treatment, and follow‐up were analyzed. The study was approved by the Hospital Ethics Committee and all patients were recruited under informed consent.

**Fig. 1:**
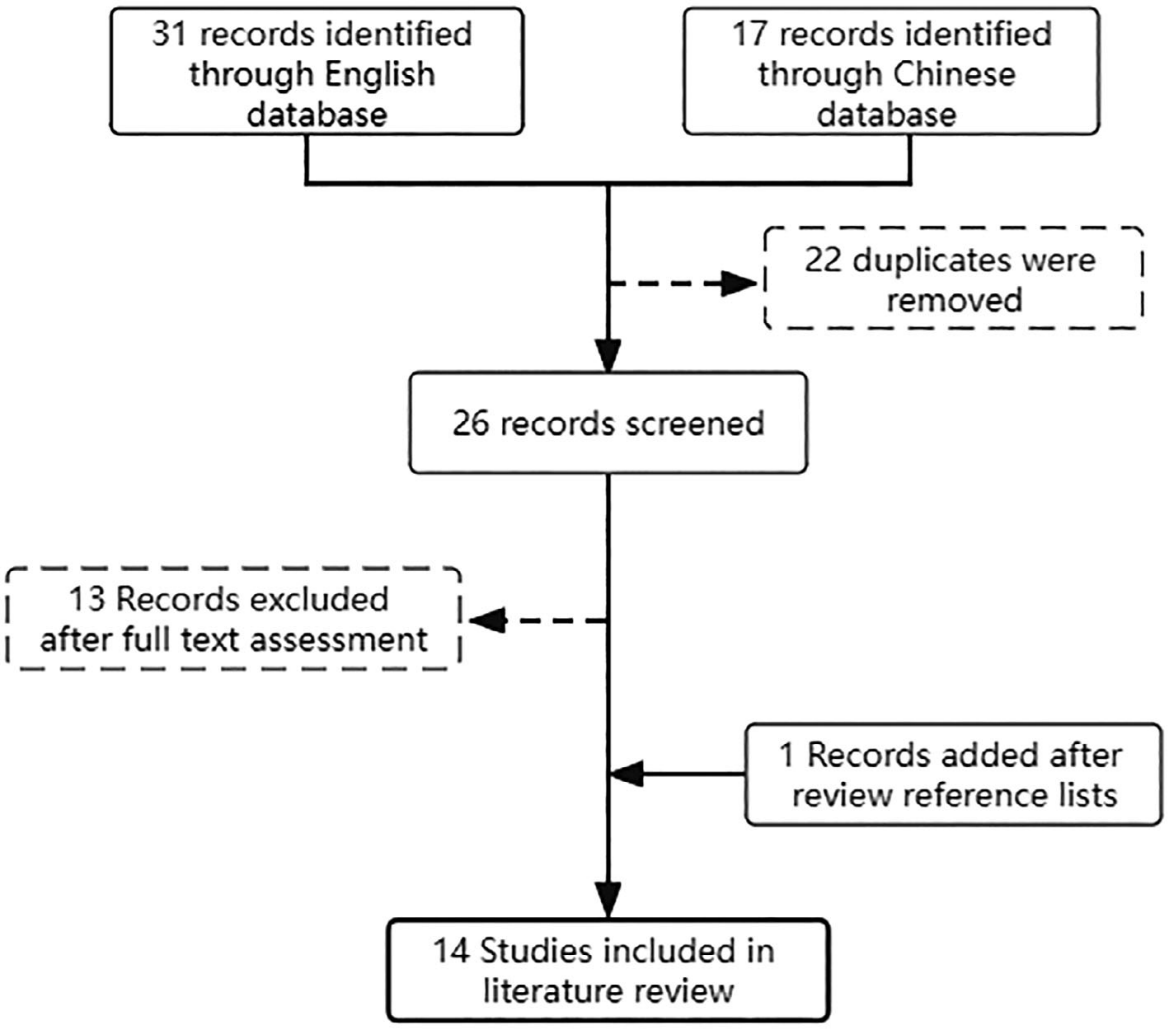
A PRISMA diagram showing the screening and selection of papers analyzed in the systematic literature review.

A systematic review of PDP was undertaken according to the PRISMA 2020 statement Preferred Reporting Items.^9^ English and Chinese articles were retrieved through PubMed, Embase, Medline, the China National Knowledge Infrastructure (CNKI) and Wanfang. Searches were conducted using the terms “postoperative pseudocyst,” “postdiscectomy pseudocyst” or “postoperative discal pseudocyst.” Two authors independently read the titles and abstracts of the relevant articles to remove repeated cases and obtain full text copies of qualified articles. The full text was read to remove articles with incomplete medical histories or treatment records. To supplement the database searches, reference lists of all of the included articles were also screened to identify additional data sources. A schematic overview of the article screening process is presented in Fig. 1.

All patient information was systematically reviewed and analyzed. All data were provided as the mean ± standard deviation. Statistical analysis was performed using a z‐test.

## Results

### General Information

Data from nine patients were included in this study and are summarized in Table 1. A systematic review of the literature identified 14 relevant articles with data from 43 cases of PDP. These articles included data from patients in Korea (28), Japan (four), China (eight), the USA (two) and India (one). A summary of the patient data is presented in Table 2.

**TABLE 1:** Summary of the patient characteristics in this study

Case	Age/Sex	Level	Direction	Pfirrmann Grade	Operation	Ag (days)	Cons (days)	Management	VAS (Pre → Post)		Follow‐up (Years)
									LBP	LP	
1	18/M	L4‐5	L	II	PETD	42	19	PETD	7 → 2	7 → 1	1.5
2	30/F	L5‐S1	L	III	PETD	20	40	MD	0 → 1	8 → 1	2.5
3	23/M	L4‐5	R	III	PETD	23	19	PETD	8 → 3	8 → 1	3.7
4	25/M	L5‐S1	L	III	PETD	23	7	TLIF	7 → 2	8 → 0	4.6
5	37/M	L4‐5	R	II	PETD	14	24	PETD	0 → 1	7 → 2	2.6
6	36/M	L5‐S1	R	III	PETD	8	28	Conservative	8 → 2	9 → 0	1.4
7	29/F	L5‐S1	L	III	PEID	16	11	Aspiration	3 → 3	8 → 0	5.8
8	27/M	L5‐S1	L	IV	MD	14	16	TLIF	0 → 0	8 → 2	8.0
9	30/M	L5‐S1	L	III	MD	27	16	TLIF	0 → 1	8 → 0	1.7

Abbreviations: Agg, Aggravation; Cons, Conservative treatment time; F, Female; L, Left; LBP, lower back pain; LP, leg pain; M, male; MD, Microdiscectomy; PDP, Postoperative Discal Pseudocyst; PEID, Percutaneous endoscopic interlaminar discectomy; PETD, Percutaneous Endoscopic Transforaminal discectomy; Pre Pro‐operation, Post Postoperation; R, Right; TLIF, Transforaminal Lumbar Interbody Fusion; VAS, Visual Analogue Scale.

**TABLE 2:** Summary of the relevant studies between 2009 and 2022

Number	Author (year)	Journal	Country	Name of PDP	Amount	incidence rate
1	Young *et al*.(2009)	SpineJ.2009 Feb; 9 (2):e9–e15	US	Postoperative annular pseudocyst	2	N/A
2	Kang *et al*.(2011)	J Korean Neurosurg Soc. 2011 Jan; 49 (1):31–6	Korea	Symptomatic Post‐Discectomy Pseudocyst	15	1% (15/1503)
3	Chung *et al*.(2012)	2012 Apr; 154 (4):715–22	Korea	Symptomatic postoperative discal pseudocyst	12	N/A
4	Yu *et al*.(2016)	Korean J Pain. 2016 Apr; 29 (2):129–35	Korea	Symptomatic Discal Cyst	1	N/A
5	Jha *et al*.(2016)	Asian J Endosc Surg. 2016 Feb; 9 (1):89–92	Japan	Postoperative discal cyst	2	N/A
6	Qiu *et al*.(2016)	Zhonghua Gu Ke Za Zhi. 2016,36 (17):1114–1120 (in Chinese)	China	Post‐discectomy pseudocyst	2	N/A
7	Prasad *et al*.(2017)	Neurol India. 2017 May‐Jun; 65 (3):650–652	India	Post‐discectomy annular pseudocyst	1	N/A
8	Shiboi *et al*.(2017)	J Spine Surg. 2017 Jun; 3 (2):233–237	Japan	symptomatic discal pseudocysts	1	0.28% (1/359)
9	Wu *et al*.(2019)	Zhonghua ji zhu ji sui za zhi.2019,29 (05):475‐480 (in Chinese)	China	Post‐operation discal pseudocyst	2	N/A
10	Manabe *et al*.(2019)	Int J Spine Surg. 2019 Feb 22; 13 (1):92–94	Japan	postoperative discal cyst	1	N/A
11	Wang *et al*.(2020)	Zhongguo jiao xing wai ke za zhi.2020,28 (09):862‐864 (in Chinese)	China	Symptomatic Postoperative Discal Pseudocyst	1	N/A
12	Fu *et al*.(2021)	World J Clin Cases. 2021 Feb 26; 9 (6):1439–1445	China	Postoperative discal pseudocyst	1	N/A
13	Li *et al*.(2021)	Medicine (Baltimore). 2021 Jan 22; 100 (3):e24026	China	Symptomatic postoperative discal pseudocyst	1	N/A
14	Xu *et al*.(2021)	Orthop Surg. 2021 Feb; 13 (1):347–352	China	Symptomatic Postoperative Discal Pseudocyst	1	1.05% (2/190)

Of the nine patients treated at our unit, seven were male and two were female and the mean age at the time of surgery was 28.3 ± 5.7 years (range 18–37 years). Percutaneous endoscopic transforaminal discectomy (PETD) was the first operation performed on seven patients and microdiscectomy was first performed on two patients. The duration of postoperative intervertebral disc cyst‐related symptoms was 20.3 ± 9.3 (range 8–42) days. Four cases reported limb pain on the affected side and five cases reported limb pain accompanied with back pain. In three cases, the PDP was located in L4/5 and in six cases the lesion was located in L5/S1. The lesions were mainly concentrated on the left side in six cases and on the right side in three cases.

**Fig. 2:**
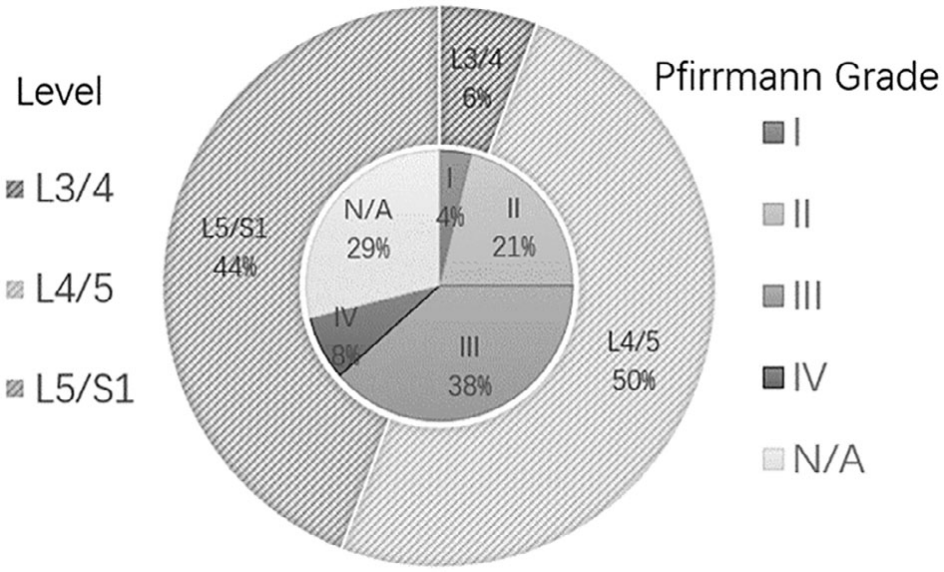
The level and Pfirrmann grades of the intervertebral discs with PDP.

A retrospective analysis of the literature identified 38 male and five female patients with a mean age of 26.3 ± 9.4 years (14–60 years). All of these patients underwent surgery for lumbar disc herniation prior to the development of PDP in MD 15 (34.9%), PETD 12 (28%) and PEID16 (37.2%). The duration of postoperative intervertebral disc cyst‐related symptoms was 35.1 ± 34.8 (range 7–240) days. There is no unified evaluation standard for the description of symptoms and signs of PDP and so these were not evaluated from the literature. Three cases had lesions in L3/4, 23 cases had lesions in L4/5, and 27 cases had lesions in L5/S1 (Fig. 2). Only 16 cases reported the direction of the lesions in which nine cases were mainly concentrated on the left side and seven cases were mainly concentrated on the right side (Table 3).

**TABLE 3:** Summary of the clinical and demographic characteristics of the patients

Item	Value
Age, years	26.3 ± 9.4 (14, 60)
Sex	
Male	38 (88.4%)
Female	5 (11.6%)
Level	
L3/4	3 (7.0%)
L4/5	23 (53.5%)
L5/S1	17 (39.5%)
Direction	
Left	9 (21.0%)
Right	7 (16.3%)
N/A	27 (62.8%)
Pfirrmann Grade	
I	2 (4.7%)
II	9 (21.0%)
III	14 (32.6%)
IV	3 (7.0%)
N/A	15 (34.9%)
Operation	
MD	15 (34.9%)
PETD	12 (28.0%)
PEID	16 (37.2%)
Aggravation time, Days	38.1 ± 37.3 (7240)
Options for PDP	
Conservative	20 (46.6%)
Interventional	4 (9.3%)
Surgical	19 (44.2%)

Note: Values are reported as $\bar{X}$ ± s (range) or number (%).

### Imaging Findings

**Fig. 3:**
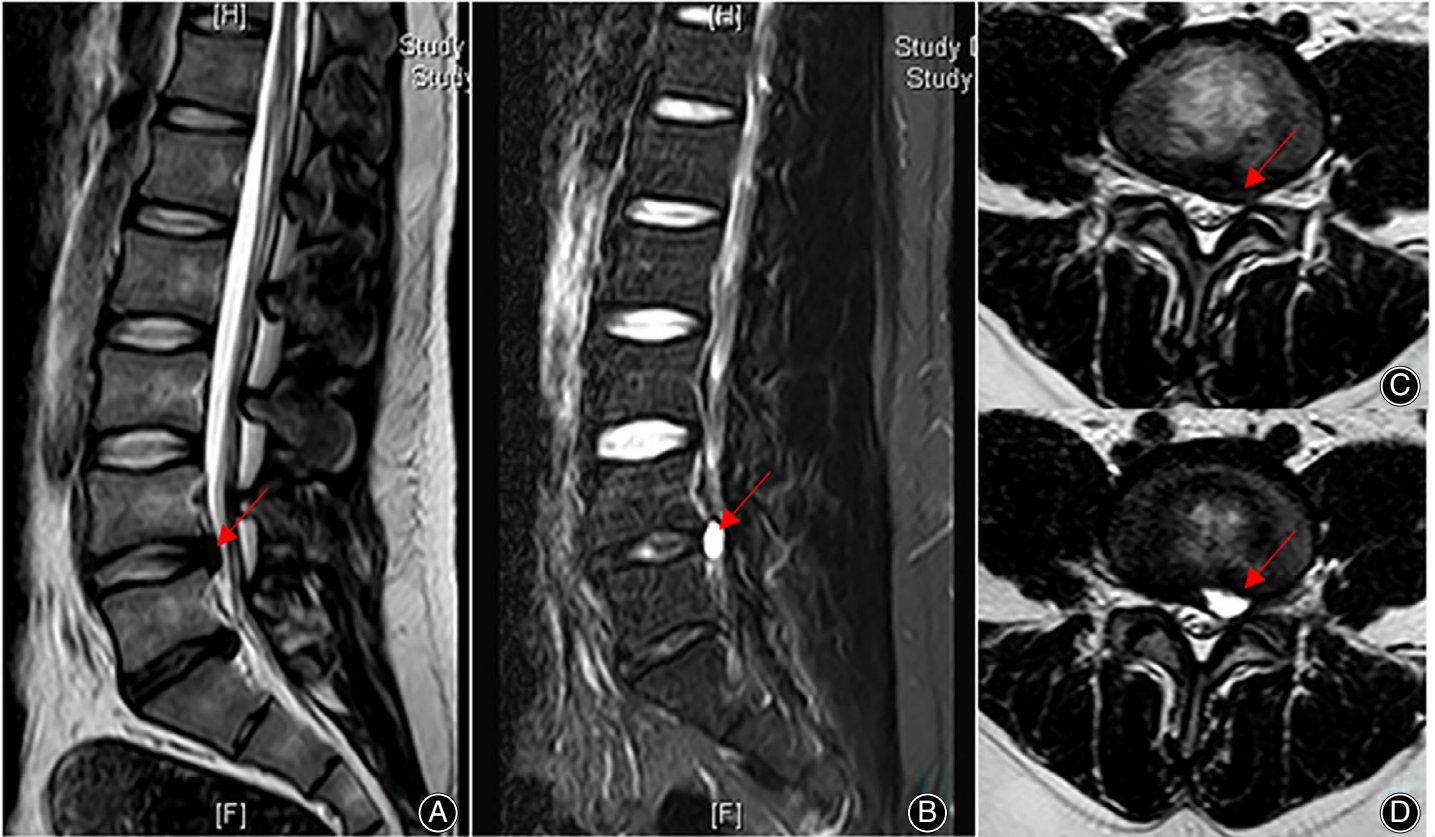
Lumbar MRI: disc degeneration (Pfirrmann grade II) and PDP. A\C: disc degeneration before lumbar discectomy, B\D: PDP (L5/S1 left).

**Fig. 4:**
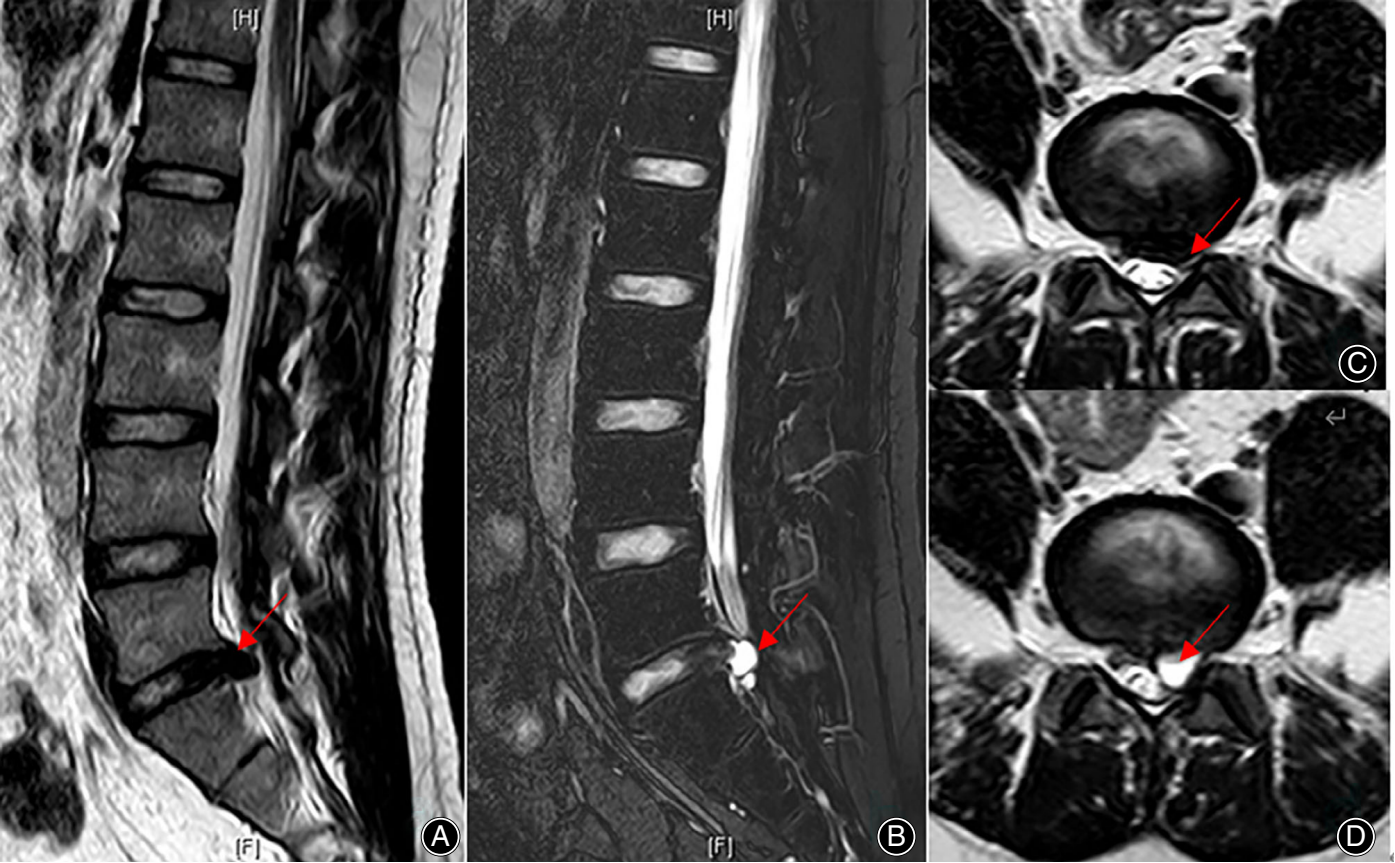
Lumbar MRI: disc degeneration (Pfirrmann Grade III) and PDP. A\C: disc degeneration before lumbar discectomy, B\D: PDP (L5/S1 left).

**Fig. 5:**
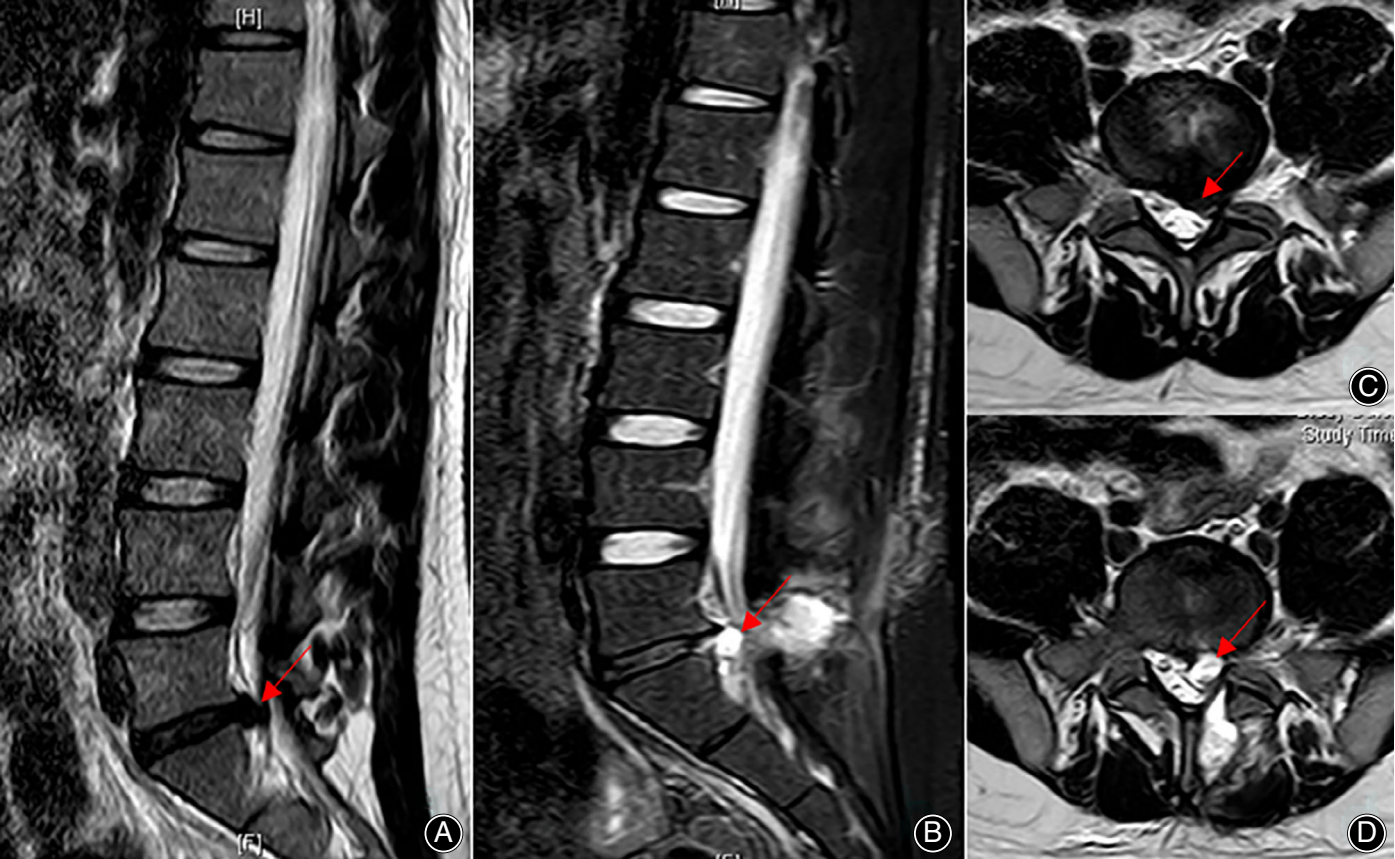
Lumbar MRI: disc degeneration (Pfirrmann Grade IV) and PDP. A\C: disc degeneration before lumbar discectomy, B\D: PDP (L5/S1 left).

In our cases, four patients underwent CT examination of the disc cysts. PDP on CT generally indicates changes in the lumbar spine after surgery and presents as the same symptoms in recurrent lumbar discs. Previous studies have reported the X‐ray or CT examination results of PDP lesions. The diagnosis of PDP is based on MRI examination with images showing signs of cysts at the surgical site and communication with the disc. From a review of the literature, all cases of PDP had a high signal intensity on T2‐weighted images and T2‐weighted fat suppression images with a low signal intensity on T1‐weighted images. Degenerative lumbar disc herniation was assessed using the Pfirrmann grading system. Of the nine cases from our center, two cases were Pfirrmann grade II, six cases were grade III, and one case was grade IV (Figs. 3, 4, 5). From the published studies, Pfirrmann grades were only reported for 28 patients of which two patients were I, nine patients were grade II, 14 patients were grade III and three patients were grade IV.

### Treatments and Outcomes

The management of PDP involves conservative treatment, interventional therapy and surgical treatment. In the nine patients from our center, seven were treated with surgery of which three patients underwent PETD and four patients underwent cystectomy. The other two cases received interventional therapy and conservative treatment.

From the cases identified in the literature, 20 patients received conservative treatment, four patients received interventional treatment and 19 were treated with surgery. The specific surgical methods included nine cases of PETD, five cases of cystectomy, and five cases did not provide a specific description of the surgical methods used. Most PDPs break when the roots are pulled apart. The capsule wall is dissected and an annular laceration is visible at the bottom of the lesion. Irrespective of the treatment strategy that is adopted, the symptoms of the patient are relieved significantly after treatment. The symptoms of all of the patients in the previous studies disappeared or were relieved during the follow‐up which was consistent with the patient outcomes at our center. The follow‐up period in this study was 1.4–8 years. A summary of the patient characteristics is presented in Table 1.

## Discussion

### Incidence Rates

Symptomatic PDP of the lumbar spine are extremely rare complications that can occur after lumbar discectomy and have mostly been previously reported as individual case reports. Kang *et al*.^6^ followed 1503 patients after surgery and found that only 15 patients had PDP. The incidence of PDP is considered to be 1% after lumbar endoscopic surgery, but all patients in the study were young and male which may have biased the results. In contrast, Shiboi *et al*.^10^ found that the incidence of PDP was 0.28% (1/359). Also, a study from China found that the incidence of PDP was 1.05% (2/190).^11^ These studies report different incidence rates for PDP in different countries with small sample sizes that may not accurately represent larger patient populations.

From the data analysis of patients treated at our center, we found a PDP incidence rate of 0.48% (9/1890). In patients with mild or asymptomatic PDP that had not been diagnosed and in patients with symptomatic PDP treated at other hospitals, some PDP spontaneously decreased or disappeared.^12^ These observations may contribute to the low reported incidence of PDP whilst the actual incidence of PDP may be higher than 0.48%.

### General Information

Our data showed that most of the PDP patients were young which agrees with previously reported cases of the disease in which patients between 14–60 years.^5^, ^10^ The patients in our study had an average age of 28.3 (± 5.7) years which compared to 26.3 (± 9.4) years of the patients in the literature. These data show that PDP patients are younger than those with discal cysts that have a reported average age of 33.5 (± 12.6) years.^13^ We also showed that PDP is more frequent in males than females with seven male and two female cases at our center. From the literature, we found 38 male and five female cases of PDP. The underlying mechanisms that result in a higher rate of PDP in young males have not been determined. These data may be related to the increased activity of males after discectomy and differences in hormone levels between men and women.

It remains unclear if the disc segments of PDP are biased. In this study, we found three cases of PDP in L4/5 segment and six cases of PDP in L4/5 segment. From the literature review, we found 23 cases in L4/L5, 17 cases in L5/S1 and three cases in L3/4. However, the number of LDH cases in each segment was not compared as different surgical approaches may bias the results. Also, we found that PDP could exist on one side of the disc or across the midline, generally on one side. These findings were related to the position of the disc herniation and may not be significant in the formation of PDP.

Current reports of PDP have mainly focused on Asian populations with the largest studies in South Korea and China. In this study, 28 cases (65.1%) were reported in South Korea and eight cases (18.6%) were reported in China. Four cases (9.3%) were reported in Japan, two cases (4.7%) in the United States and one case (2.3%) in India. These data suggest regional differences in the occurrence of PDP.

PDP usually appears during the early stage of lumbar discectomy but the exact timing of PDP remains controversial. Chung *et al*.^7^ calculated the formation time of PDP in 12 cases according to the examination time of PDP confirmed by postoperative MRI indicating that the average time of PDP complications after PELD was around 1 month. Using the same method, Wu *et al*.^14^ analyzed 39 cases of PDP and reported an average PDP formation time of 61 days. Considering that some patients did not receive MR examinations in time after presenting with PDP, we believe that the occurrence of PDP is earlier than has been reported.

We analyzed the time of postoperative symptoms in 43 patients from a review of the literature and found that the time to postoperative symptoms related to disc cysts was 38.1 ± 37.3 (7240) days and 20.3 ± 9.3 (range 8–42) days in the nine patients in our unit. The onset time of symptoms in patients can more accurately reflect the formation time of PDP compared to the time of symptomatic PDP confirmed by MRI. However, some PDP patients may be asymptomatic in the early stages and so the formation time of PDP may be earlier.

PDP has very similar characteristics to discal cysts except for the history of operative treatment for LDH.^10^ The patients treated at our center mainly manifested with recurrent lumbar and lower limb pain after lumbar discectomy. Lower limb pain was the main symptom that simulated recurrent LDH.

### Pathogenic Mechanisms of PDP

The pathogenesis of PDP remains unclear and several potential mechanisms have been proposed. Young *et al*.^5^ proposed that PDP results from intervertebral disc inflammation which stimulates the epidural space to form a pseudocyst in the intervertebral disc. Surgical removal of the intervertebral disc and pseudocyst is retained. Physical activity results in mild degeneration of the nucleus through the annular crack pump into the membrane formation leading to the formation of the pseudocyst. This hypothesis is supported by several research groups.^6^, ^7^, ^14^ Kang *et al*.^6^ believed that heat generated by RF cauterization or laser coagulation during endoscopic surgery promoted the development of intervertebral disc inflammation. This process may explain the increasing number of reports on PDP with the development of endoscopic technologies.

A further hypothesis involves the emergence of the nucleus pulposus after the formation of membrane structures connected by a fibrous ring. During the operation, the emergence of the nucleus pulposus in front of the rear area is retained. The nucleus pulposus form in the cystic cavity formation resulting in postoperative bleeding and so liquid accumulates in the cavity to form a pseudocyst.^15^ This process is not consistent with previous reports that nucleus pulposus tissue was not found in the PDP capsule wall structure and so the hypothesis needs further validation.

Other studies have suggested that PDP and disc cysts are homologous diseases with a conserved pathological mechanism. PDP is caused by delayed bleeding at the discectomy site, and TCM‐derived annular injury in discectomy may accelerate the pathological progress of DC.^16^, ^17^ The current mechanism of DC formation is also controversial. A case of PDP secondary to a previous infectious discitis was recently reported^18^ which did not discuss the role of infection in the formation of PDP. Based on a review of the literature, only Young *et al*.^5^ found that PDP was excluded as a possible cause of infection during diagnosis and so the role of infection factors in PDP requires further investigation.

### Imaging Features

Intervertebral disc cysts are mostly found in young people with mild intervertebral disc degeneration. MRI shows a high signal on T2‐weighted images and a low signal on T1‐weighted images.^7^ In this study, Pfirrmann grade was used to evaluate the degree of intervertebral disc degeneration. Our data show that patients mostly had Pfirrmann grade II or III lesions. The nine cases in our center were mainly grades II (two cases), III (six cases) and IV (one case). These data support the mechanism of PDP formation proposed by Young *et al*. and that mild degeneration of the intervertebral disc may be a risk factor for PDP. However, this hypothesis requires further investigation.

### Management

Conservative treatment should be considered in patients with less severe symptoms as smaller cysts may disappear over time.^6^, ^12^, ^17^, ^19^, ^20^ Conservative treatment includes complete bed rest, analgesia, and physical therapy.^7^, ^19^ In one case, antibiotics were considered to treat discitis as surgery did not control infection.^19^ There is a lack of evidence to support the use of antibiotics in the treatment of PDP. In this study, 20 (46.6%) patients received conservative treatment. However, no clear conclusions could be drawn concerning the duration of conservative treatment. All nine patients in our unit received conservative treatment which lasted for 20 ± 9.2 (8–43) days. One patient was lost to follow‐up after 28 days of conservative treatment. We believe that at least 1 month of recovery should be given before interventional treatment or surgical intervention unless the patient has severe symptoms.

The long‐term use of medication can negatively impact liver and kidney function, and prolonged bed rest may cause muscle atrophy. For patients who do not want to be treated again, interventional therapy under the guidance of DR or CT can be used.^21^ Interventional therapy involves needle aspiration and injections of either imaging contrast agents for diagnosis or drugs (steroidal, xylocaine with dexamethasone, ozone) for treatment. Diseased tissues can be removed and cultured to rule out infections.^5^, ^21^, ^22^ In the cases in the literature, four patients (9.3%) who received conservative treatment were lost during follow up indicating that interventional treatment is an effective treatment option for PDP.

After conservative OR interventional treatments, some patients had poor responses and required surgery.^19^, ^22^ Surgical treatment options for PDP include microscopic partial hemilaminectomy, transforaminal endoscopic discectomy, endoscopic discectomy, percutaneous endoscopic lumbar discectomy (PELD), percutaneous endoscopic discectomy and cystectomy.^6^, ^10^, ^11^, ^22^ From the cases reported in the literature, 19 patients (44.2%) underwent surgery which successfully relieved all symptoms at the last follow‐up. For surgery to remove the disc, studies suggest that microsurgical cystectomy without corresponding discectomy is an effective surgical treatment for lumbar discal cysts^23^ yet this approach has not been explored in the treatment of PDP. Among the seven patients who received surgical treatment in our unit, only cysts were removed in four cases, and discectomy and corresponding segment fusion were performed in three cases. Surgery treatment can completely remove PDP to quickly relieve symptoms after surgery, however, PDP can recurrence after surgery.

Studies have reported no significant differences in treatment outcomes following surgery or conservative treatment for symptomatic PDP.^6^ Surgical treatment should be performed with caution when conservative treatment is ineffective. Also, the surgical method should be carefully considered based on the condition of the individual patient and surgical expectations.

### Conclusion

PDP is a rare complication that can occur after lumbar discectomy. PDP occurs in Asian males with mild intervertebral disc degeneration 1 month after discectomy. The pathogenesis of this disease is unclear and it may be related to infections. Treatment should be selected according to the specific situation of patients. In cases where conservative treatment is necessary, surgical treatment should be carried out with caution.

## Author Contributions

Zhenlu Cao wrote the manuscript. Yanan Cong collected the data and reviewed the manuscript. Chuqiang Yin, Yuelei Wang, Zhichao Wang and XiaoWei Liu participated in the surgery and revised the manuscript. Ting Wang was the lead surgeon and revised the manuscript.

## Conflict of Interest Statement

The authors declare that they have no known competing financial interests or personal relationships that could have appeared to influence the work reported in this paper.
